# Person-Centered Healthcare Practice in a Pandemic Context: An Exploration of People's Experience of Seeking Healthcare Support

**DOI:** 10.3389/fresc.2021.726210

**Published:** 2021-09-01

**Authors:** Eleanor Curnow, Vaibhav Tyagi, Lisa Salisbury, Kim Stuart, Barbara Melville-Jóhannesson, Kath Nicol, Brendan McCormack, Jan Dewing, Ruth Magowan, Olivia Sagan, Cathy Bulley

**Affiliations:** ^1^Division of Dietetics, Nutrition and Biological Sciences, Physiotherapy, Podiatry and Radiography, School of Health Sciences, Queen Margaret University, Edinburgh, United Kingdom; ^2^Division of Nursing and Paramedic Science, School of Health Sciences, Queen Margaret University, Edinburgh, United Kingdom; ^3^Research and Knowledge Exchange Development Unit, Queen Margaret University, Edinburgh, United Kingdom; ^4^Long Covid Scotland, Edinburgh, United Kingdom; ^5^Division of Psychology, Sociology and Education, School of Arts, Social Sciences and Management, Queen Margaret University, Edinburgh, United Kingdom

**Keywords:** Person-centered Practice Framework, person-centered practice, long-covid, support, illness, COVID-19

## Abstract

**Background:** The recent COVID-19 pandemic increased pressure upon healthcare resources resulting in compromised health services. Enforced national lockdown led to people being unable to access essential services in addition to limiting contact with social support networks. The novel coronavirus, and subsequent condition known as long covid were not well-understood and clinicians were not supported by existing guidelines or pathways. Our study explored people's experiences of healthcare during this period with a person-centered “lens.”

**Methods:** Ninety-seven people participated in our online survey about their experiences of the pandemic, particularly while socially isolated and their experiences of healthcare. Following completion of the survey, 11 of these participants agreed to further semi-structured interviews to explore this further in their own words. Interview conversations were transcribed, checked; together with the responses to open questions in the survey. The data were then analyzed thematically by members of the research team. We conducted framework analysis from a post-positivist perspective, using the Person-centered Practice Framework to explore participants' experiences.

**Results:** There were few examples of people describing person-centered care. People experienced barriers to accessing support, and negative experiences of care that represented complexities enacting person-centered care at each level of the framework (processes, practice environment, prerequisites, and macro context). These barriers were influenced greatly by the pandemic, for example, with health professionals being harder to access. Some experiences related to the ways in which health professionals responded to the context, for example, positive examples included active listening, recognition of people's experiences, seeking to find out more, and engaging in collaborative problem-solving.

**Discussion:** People want to feel heard, supported to navigate healthcare systems, source trustworthy information, find appropriate services, and collaborate in learning and problem-solving with healthcare professionals. There have been enormous challenges to the provision of healthcare throughout the pandemic. Moving forward is crucial with emphasis on overcoming barriers to person-centered healthcare. This should focus on steps now and also in planning for the possibility of further rapid changes in the demand for and provision of healthcare.

## Introduction

In early 2020 the COVID-19 pandemic introduced unprecedented pressure on healthcare resources. Enforced lockdown further reduced access to social supports. This resulted in changes to the patient care environment, healthcare policies, and service availability. People experienced social isolation and were forced to manage illness without the benefit of social support, or other services they would normally receive. In this context there have been immense challenges to enacting person-centered care which has affected many people living with long-term conditions and developing new conditions. It is therefore, crucial to explore this further.

Long-term conditions are defined by their duration (lasting a year or more) and their impacts on people's lives due to the need for ongoing care (1). Before the COVID-19 pandemic they were the leading cause of mortality globally, placing huge demands on healthcare (2, 3). Support has been disrupted, however, due to the necessary focus on the COVID-19 pandemic. This has negatively impacted people living with long-term conditions, due to impacts of social distancing on lifestyle behaviors, mental health, changes to routine management, new diagnoses, medication adherence and progression of condition (4). An international online survey conducted in April 2020 (5) found that 202 health professionals from 47 countries felt that routine care for people with long-term conditions had been negatively impacted, requiring change from routine care to virtual communication. A fifth of respondents also reported negative impacts on their patients' mental health. There has been creative use of healthcare technologies, but this does not enable necessary care for all. Evidence from earlier epidemics and health emergencies supports substantial risk of a “post-pandemic double burden of disease” due to neglect of people living with long-term conditions (6). It is crucial to re-focus on public health and support people living with long-term conditions in order to avoid increased morbidity and mortality.

In addition to the impacts of the pandemic on existing health conditions, we have seen the emergence of long covid in around 10% of people who have had COVID-19 in the UK. The Office for National Statistics (7) estimated that on 6/3/2021 1.1 million people in private households (1.7%) in the UK had self-reported long covid, with almost 18% of these people feeling that their “ability to undertake their day-to-day activities had been limited a lot.” Prevalence rates were greatest in people of working age, women, people living in deprived areas, and people with “a pre-existing, activity-limiting health condition.” This list includes several characteristics commonly seen as intersectional influences. About half of those who self-reported long covid contacted the NHS but were not hospitalized when they developed COVID-19 symptoms, while only around 8% were admitted.

Symptoms of long covid reflect involvement of different body systems and fluctuate over time (4). The most prevalent self-reported symptoms that persisted for at least 5 weeks after the assumed date of COVID-19 infection included fatigue, cough, headache and myalgia. Prolonged symptoms were more common for females, people aged 35–49 years, and were greatest for health and social care workers. Other reported symptoms of long covid include shortness of breath, chest pain, headaches, neurocognitive difficulties, muscle pains and weakness, gastrointestinal upset, rashes, metabolic disruption, thromboembolic conditions, and depression and other mental health conditions (8). A recent dynamic NIHR review found long covid to be a debilitating condition that affects multiple body symptoms and may involve active disease that requires ongoing monitoring. They synthesized studies proposing that Long Covid includes different syndromes, such as ongoing active COVID-19, development of a new condition such as post-intensive care syndrome, post-viral fatigue, or post-traumatic stress, and negative impacts on a long-term condition that the person was already living with. This will require further research, but the impacts are becoming clear. In their own survey, the reviewers found that over 70% of survey respondents felt it affects family life, with 39% finding it hard to care for dependents, and 80% found it was affecting their ability to work (9). An earlier dynamic review by the same team concluded that greater understanding and support for people to recover is crucial to reduce substantial long-term psychological and social impacts. Likely disproportionate impacts of long covid on people with lower incomes and in minority groups are highlighted (9, 10). In this situation, it is crucial to consider how person-centered practice is, or is not, being enacted.

There is substantial evidence to support policy which advocates a person-centered approach as core to healthcare delivery for people living with long-term conditions (2, 11). The World Health Organization (WHO) places a priority on re-orientating health services around people rather than health conditions and institutions. The WHO framework on integrated people-centered health services envisions health services which are provided according to peoples' needs, and respect their preferences, in addition to being safe, effective, timely, affordable, and of sufficient quality (2). Person-centered practice is based upon core values that include shared autonomy; therapeutic caring; commitment to healthfulness as process and outcome; respect for a persons' individual abilities, preferences, lifestyles and goals; and the demonstration of mutual respect and understanding (12). There is emphasis on equal partnerships between people seeking care and the providers of care, in all stages of the process to ensure that the person's needs are met (13). These principles are reflected in UK health policies. For example, NHS Long-Term Plan (14) promotes the principle of people gaining control over their own health, and the personalization of care.

**Figure 1:**
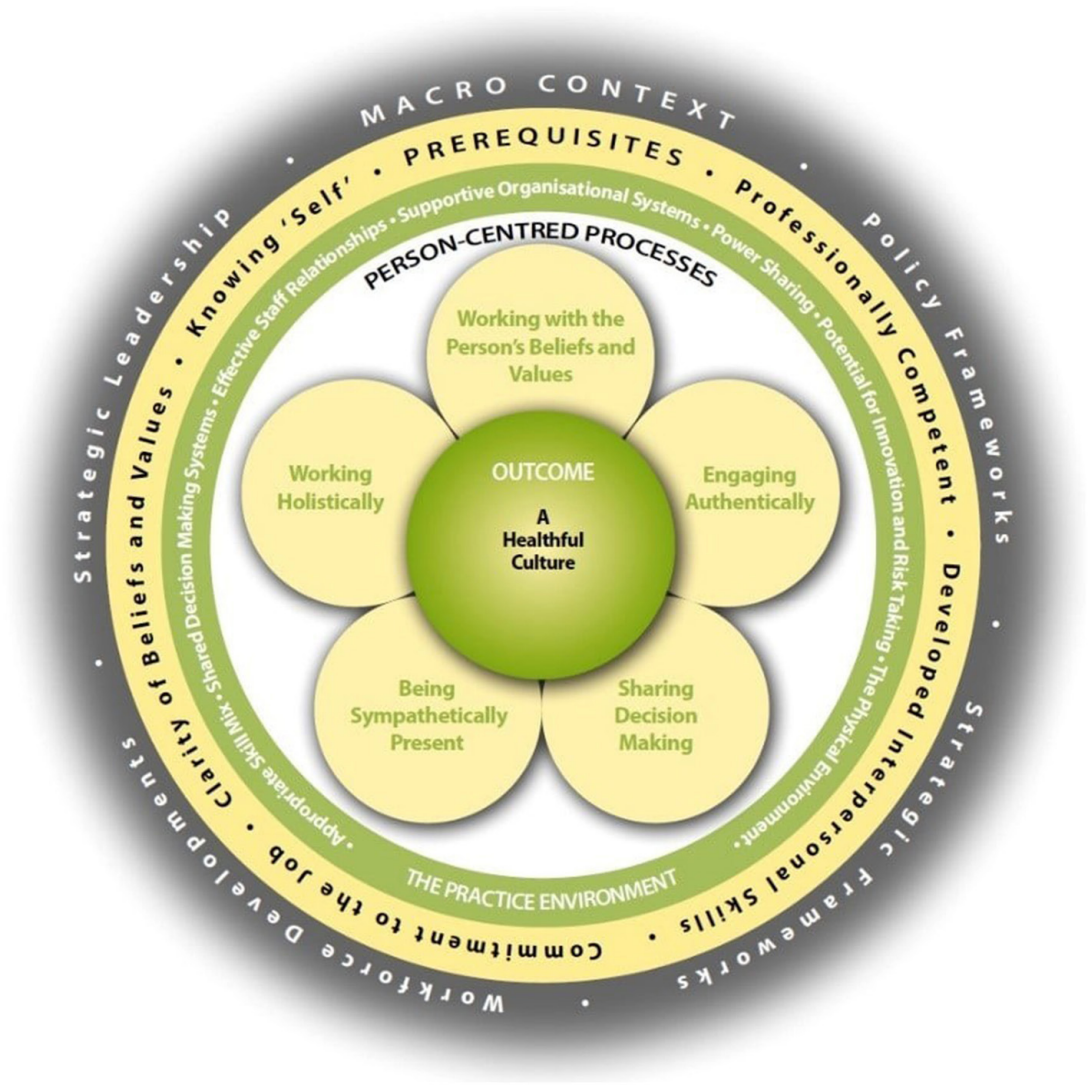
The Person Centered Practice Framework (21). Reproduced with permission.

It is important to operationalize principles and values to find ways of ensuring their implementation in practice. Use of a person-centered practice model has been shown to contribute to enhanced outcomes for service users, better use of resources, decreased costs and increased satisfaction with care (15). Several conceptual models have been developed in different contexts and with varying components. Four examples include those of Mead and Bower (16), Hobbs (17), Morgan and Yoder (18), and McCormack et al. (19). These were discussed by Dukhu et al. (20) in relation to their similarities and differences. While there are notable similarities in underlying contexts, it is notable that several have quite a specific focus, for example on doctor-patient relationships (16) and on nursing practice (17, 18). The Person-centered Practice Framework (19) has developed from an early focus on nursing, to broader healthcare practice, with iterative development over years to the current version (see Figure 1).

The Person-centered Practice Framework (PcPF) describes the ultimate outcome as a “healthful culture.” The Framework provides analysis of the complex aspects of practice that interact to produce this, with the most direct impacts being from “person-centered processes” that include: working with the person's beliefs and values, engaging authentically, sharing decision making, being sympathetically present, and working holistically. These processes are thought most likely to be enacted if the Practice Environment supports them, through: enabling power sharing, enabling innovation and risk taking in the interests of the person, supportive organizational systems, appropriate physical environment, skill mix, shared decision-making systems and effective staff relationships. The likelihood of these aspects of practice being in place is then influenced by prerequisites, requiring team members that are professionally competent, who have developed interpersonal skills, commitment to the job, clarity of beliefs and values, and who “know self.” This all takes place within a wider “macro context” which involves policy, strategy, workforce development and strategic leadership (19). The PcPF provides a valuable operationalization of different influences on a person's experience of healthcare, as well as of the experiences of people providing healthcare, which can be used to analyze current practice, and has been used to do so in many contexts (21–24).

Clearly, we are in a time of enormous change in healthcare demand and delivery for people with many long-term conditions, including long covid has emerged. The second NIHR dynamic review of long covid concludes that there is a need for joined-up provision across both specialties and between primary and secondary care, requiring a “multi professional workforce strategy” (9). It is a crucial moment to explore how such workforce strategy and service development can incorporate person-centered principles, and how that might impact on patient outcomes as well as wellbeing of health care providers. Our study moves into this discussion, bringing voices of people with lived experience into the spotlight in the hope of informing the journey forward. In order to develop services which, support people through this type of situation, it is important to firstly understand the impact of healthcare services, operating in a pandemic situation, on the wellbeing of participants. This study aims to use the PcPF as a lens through which to evaluate people's experiences of healthcare services during the pandemic in order to inform a re-focus on supporting people with long-term conditions in a person-centered manner.

## Materials and Methods

This study formed part of a multi-stage pragmatic research study investigating the experiences of people who had been ill while feeling isolated; in order to develop insights into what support people require during their recovery and how they access this support.

Results were used this to generate recommendations for policy and planning relating to service provision.

**Figure 2:**
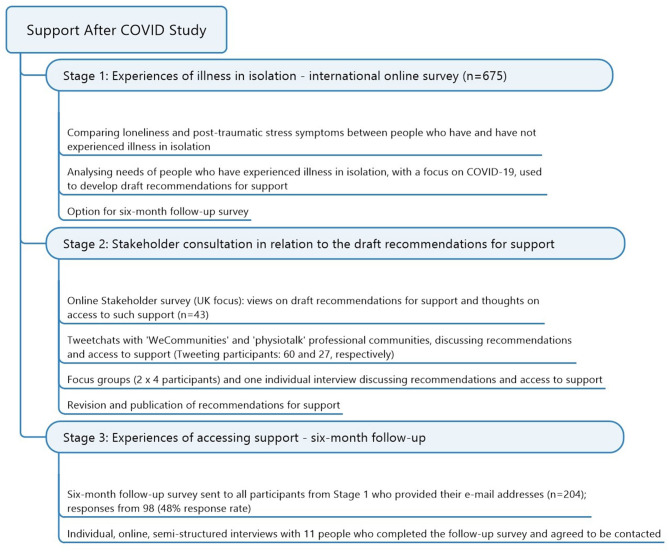
Summary of all stages of the multi-stage “Support After Covid Study”.

The overall study included the stages summarized in Figure 2, are explained below:

1. Exploration of people's experiences of illness while feeling isolated and their perceived support needs through an international online survey in order to develop draft recommendations.
2. Evaluation of draft recommendations through different forms of stakeholder consultation to refine the recommendations.
3. Exploration of changing experiences of accessing support 6 months after the initial survey through a follow-up survey and online qualitative interviews.

The first stages of this study have previously been reported (25). We are now reporting the third stage, and will provide some methodological detail from the first two stages because they are interlinked. All aspects of the study were reviewed by Queen Margaret University Research Ethics Committee.

### Stage 1

The initial international online survey was conducted from 14th July to 31st August 2020 (7 weeks), using Qualtrics Online Survey Software (2020 Qualtrics LLC). It was circulated through a wide variety of networks and promoted using social media (Twitter handle @SupportAfterCovidStudy). We sought responses from people who had not felt socially isolated as well as those who had, for comparison. We also included people who had felt ill and who had not, as well as those who had felt ill due to COVID-19. Further inclusion criteria were: aged 18 or over; and able to complete the survey in English. In addition to distributing emails, tweets or online postings using social media, a snowball sampling method was used. Survey completion took about 15–20 min.

You can view the survey online at: https://26205d15-9a75-4aa4-b2d2-185aee599a78.usrfiles.com/ugd/26205d_377147626804489d92c2aec43e3a6bd4.pdf.

Respondents who thought they had experienced COVID-19 were asked about this, including when, what support they received, and experiences of hospitalization. Questions enquired about recovery time, whether people were still experiencing symptoms, what these were, and their impact. Finally, all survey respondents were asked about the help required to support their health and wellbeing.

Draft recommendations were developed from the data (25).

### Stage 2

The Stakeholder consultation was UK focused and aimed to explore the appropriateness, completeness, and achievability of the draft recommendations. This process is reported fully elsewhere and used multiple strategies (summarized in Figure 2) to source the views of varied stakeholders, including people with lived experience, service providers and others involved in designing/funding services.

### Stage 3

We are reporting on the final stage of the study—exploring experiences of accessing support 6 months after the initial survey through a follow-up survey and online qualitative interviews. The same survey structure as stage 1 was used, and adapted in order to:

- explore whether anyone had developed new illness, and whether they thought this was due to COVID-19, since the previous survey;
- explore people's experiences in relation to each of our recommendations—we presented the form of support and asked for people's responses on a Likert scale relating to how much they thought they needed each form of support; and if needed, how easy it was to access and how helpful it had been;
- enable open responses in relation to any other types of support or contributions people wanted to make.

You can find this survey at: https://26205d_2085f490f1594d56b4c12e3ec87ab197.pdf~(usrfiles.com).

The online survey was distributed electronically via email to people who had responded to a similar survey 6 months previously and had granted permission to be contacted again for this purpose.

Following completion of the second online survey, respondents were asked if they were happy to be contacted in regard to participating in an interview which would explore their experiences in greater depth. Survey respondents who shared their contact details and agreed to be contacted were then provided with additional information regarding the interview process and content by email. They were asked to respond to this email if they still wished to participate in an interview. The interviewer (EC) then contacted each of the 11 participants by email to arrange a convenient time for the interview, and to agree a suitable online platform or phone number for the interview to take place. The interviews took place during March and April 2021.

Interview questions included asking participants:

- to describe their health and wellbeing since the start of the pandemic;
- how their health and wellbeing had changed over time, and the consequences this had on their life;
- about the support they have sought, how they found support, and how helpful this support was for them;
- things they would have liked to be different;
- key messages they wished to share with people making decisions regarding supporting people during the pandemic.

Interviews were recorded, and then transcribed verbatim. The interviewer (EC) then checked the transcription for accuracy.

A framework analysis method based on the Person-centered Practice Framework (21) was used for the thematic analysis of transcribed interview data and open response survey data (26). This method was selected once the homogeneity of the issues discussed within the semi-structured interviews and survey was recognized. The PcPF is intended to create a shared understanding of the aspects of healthcare which support healthful outcomes and was thought to be a valuable tool to explore people's experiences. Open coding was used to identify substantive themes within the data, which were then charted and summarized into the identified categories. Interpretation of the data involved identifying findings which illustrate how healthcare services responded to the demands of the pandemic, and interpreting aspects of the response through the use of the PcPF.

## Results

### Participants

The 11 interview participants were aged from 46 to 75 years. They all described themselves as British, English, Northern Irish, Scottish, or Welsh; resident in the United Kingdom; with a white background. Ten participants identified themselves as female, and one as male. Four participants did not feel more isolated than before the pandemic, and seven participants did feel more isolated. All of the participants reported being unwell during the pandemic and had experienced a range of new and ongoing health issues including COVID-19, long covid, heart attack, asthma, mixed affective disorder, depression or low mood, over-active thyroid, urology complications, Addison's disease, diabetes, and hypothyroidism.

Ten participants reported that long covid had impacted their lives greatly. One year on from contracting COVID-19, these participants were still unable to work, care for their families, or participate in activities which they previously enjoyed.

One participant had been shielding due to a number of ongoing medical conditions including diabetes, vitamin B12 deficiency, an abscess and a benign tumor.

Interview participants were recruited from the ninety-seven people who responded to the online survey. Demographic characteristics of survey respondents are presented in Table 1. Survey respondents were most likely to be female (*n* = 77), and living in the UK (*n* = 90).

**Table 1:** Demographics of online survey participants.

		***N***
**Gender**	Male	17
	Female	77
	Did not wish to state	2
	Missing	1
**Country of residence**	UK	90
	Finland	1
	Germany	2
	Ireland	2
	Norway	1
	Missing	1
**Living situation**	Living alone	17
	Living with one person	46
	Living with more than one person	33
	Missing	1
**Age**	Mean = 53 years 4 months	
	Range = 20–87 years	
	Missing = 2	

*N = 97*.

### Data Themes

Themes identified during the analysis of the data will now be described. These themes included;

Theme 1. “Participant experience of illness.” All participants had been unwell during the pandemic and were asked to provide details of their illness.
Theme 2. “My GP has been incredibly supportive with knowing nothing.” This theme acknowledges the supportive nature of person-centered processes such as engaging authentically, and being sympathetically present, particularly during a period where understanding of long covid was limited, and access to support was often restricted by lockdown.
Theme 3. “A brick wall.” Participants described the challenges they faced in attempting to understand their illness and access the support they need for recovery. Healthcare organizational systems were affected by policies introduced to mitigate the pandemic, staff shortages, and were often slow or unable to respond to new information regarding long covid. Participants therefore faced struggles which included having to educate health professionals about their condition in order to access services, and negotiating narrow referral criteria developed before the advent of long covid.
Theme 4. “It's real to you.” Rather than engaging authentically, participants felt that health professionals sometimes made decision based upon the participants' personal characteristics rather than the symptoms they were experiencing. Participants described professionals making references to their weight, gender, or suggesting long covid symptoms such as breathing difficulties were the result of mental health problems, or personal trauma.
Theme 5. “Patient as Expert.” Participants were unable to access the knowledge and skills they needed to recover, and developed understanding of their condition through personal research and support groups.
Theme 6. “Making Connections.” Participants discussed the benefits of social support to their quality of life, and how lockdown measures which restricted social contact, also impacted their health.
Theme 7. “Thoughts about the Future.” Participants felt let down by the health services they received and hope that lessons will be learned from their experiences to improve services in the future. Participants suggest that their health has been adversely impacted by the delays in accessing support.

Each theme is explained in further detail with illustrative interview data.

#### Theme 1: Participant Experience of Illness

Nearly all the study participants had experienced long covid (*n* = 10). They described long-term problems with fatigue or breathlessness which disrupted their daily activities and meant they were unable to return to work, exercise or complete household tasks. Participants with long covid, had all contracted COVID-19 at the beginning of the pandemic, meaning that at the time of this study, they had been experiencing symptoms for over 1 year.

One participant stated that for over a year, she had been:

“*Short of breath, exhausted, pains in muscles, in joints, migraine. I had diarrhea for a couple of months, I get stomach problems. I get about 50 different symptoms of all sorts of things like losing hair, and just like not just a bit of dry skin its flaky skin. Random pains, and different sensations in the body. Inability to keep a thread of thought properly*.”… “*There have been times when I haven't been able to work out how to make a cup of tea. I've had to look at the kettle and work out that I've got to put water in it; it's been as bad as that. And even now I struggle with working out how to do things. And my work, when I do try and make some notes, that I can't spell anything, I read it back and there's words missed out which doesn't really make much sense*” (Participant 7).

Another participant described the symptoms she had been experiencing for over a year:

“*I've actually been quite poorly with various problems. My breathing is still affected and although I've got a clear chest X-ray and no sort of indicators on the blood tests or anything. And I've got a lot of the sort of classic symptoms of long covid now: sort of extreme fatigue, aches, pains, headaches. A lot of the weird stuff that, I don't know, like crawling skin, numbness, tingling, weird sensations like rashes, mouth ulcers, a lot of other stuff. Burning on your skin, I don't know, all that kind of stuff* ” *(*Participant 5).

Symptoms meant she was unable to work and was dependent upon her teenage son to carry out household tasks such as shopping. Additionally, as she had been unwell for 1 year and was still unable to consider returning to work, she was facing uncertainty regarding the continuation of her employment, and her sick pay would soon end.

#### Theme 2: “My GP Has Been Incredibly Supportive, With Knowing Nothing”

Participants in this study discussed the relationship they had with their GP, who was seen as important in providing a diagnosis which facilitated access to other healthcare services, and in many cases was also seen as a source of support. As long covid was a new diagnosis, some participants found themselves forming a new relationship with their GP where they worked together to understand the condition. Clinicians who openly acknowledged the limitations of their knowledge regarding this new condition, were more likely to be trusted by participants:

“*The ones that said I don't know, I've never seen this before, we're learning as fast as we can, those are the ones that I trusted the most*” (Participant 10).

Participants appeared to be understanding of clinicians' limited knowledge regarding long covid, and appreciated healthcare professionals who took time to listen and made an attempt to provide support:

“*My normal GP has been very sympathetic and kind, but she's not able to do anything. And she's not been keeping up with the developments over the last few months within long covid, as people are starting to understand a bit more about it. She's not really kept up with that*” (Participant 6).

“*So, she's very helpful where she can be. But she's also loathed to start trying to offer advice where she doesn't know; which is probably good*” (Participant 2).

“*Key listening skills are phenomenal, really, really important and should be encouraged*” (Participant 4).

Interpersonal skills and taking time to demonstrate understanding of the patient experience were remembered and appreciated:

“*I was at an appointment a couple of years ago and a doctor came out to see me and he said to me, I just want to say to you that I've just spent half an hour reading your notes. And he said, you have so much to deal with, you've just so much to cope with. And I burst into tears. And I said thank you just for acknowledging that, that is a lot*” (Participant 8).

Some participants found the relationship they had with their GP, or other health professional, was a source of support. In some cases, the GP knew the participant well-enough that they noticed when the participants' condition worsened and was able to offer advice to attend the hospital or seek other help:

“*He knows my job, he knows what I'm normally like, and that's been really helpful*” (Participant 7).

This was only possible, however, when participants were able to see the same health professional regularly. Other participants were frustrated by being unable to be seen by the same doctor, or were only able to access medical advice via phone call, by anonymous e-consultations, or in some cases they struggled to be seen at all.

#### Theme 3: “A Brick Wall”

As a result of pandemic restrictions there were difficulties in accessing healthcare services:

“*I felt like GPs had basically shut down, they really weren't able to operate in the way they were used to because of all the things they had to put in place*” (Participant 9).

Long covid clinics in some areas appeared to be set up rapidly, without providing staff with the training required to provide a useful service:

“*I spoke to a physiotherapist a few times; she wasn't a specialist… she said normally I deal with old people leaving hospital*” (Participant 7).

Soon afterwards, the physiotherapist rang to say:

“*I'm not allowed to speak to you anymore, this little team is being shut down*” (Participant 7).

Participants were also concerned that this wait for treatment had prolonged their illness or exacerbated their condition:

“*You wonder if we had got seen if we would have gone into this or been so bad*” (Participant 12).

Some participants felt that healthcare services failed to provide support if their symptoms were not validated by a formal covid diagnosis confirmed by a positive clinical test:

“*The symptoms could be looked at more than just saying its COVID, after COVID we have no idea what to do. And obviously during the initial COVID goings on, nobody was seen. So, we weren't seen, we weren't tested, nothing was given to us. So, they don't… that's why people were dismissed because they had no tests. And it's just like there needed to be a better… like it's a postcode lottery or a GP lottery, how you get treated*” (Participant 6).

Participants sometimes found that their pattern of symptoms did not match the early diagnostic information for COVID-19 or long covid, and this also meant that they were unable to receive a formal diagnosis which would give them access to support services:

“*I would come off the phone in floods of tears, because it was so frustrating and to be told you can't have a viral infection because your bloods are all fine, because I didn't have the three symptoms which the government are still putting out there*” (Participant 3).

Participants provided other examples of being unable to access services due to narrow or complicated criteria which were not always clear or helpful:

“*Well contacting the GP is extremely difficult. You'd have to ring at half past eight in the morning, which is about all I—the one time I actually am sometimes asleep—and book a telephone conversation. And that means you've got to do it on the day when the right GP is there, and you probably don't know what that is because they keep changing days and working at the other practice eight miles away which they've linked up with. And then you have to have a telephone conversation for them to decide whether they need to see you. And sometimes the energy that takes is too much*” (Participant 2).

Another participant was unable to attend the fatigue clinic as her fatigue was too severe:

“*The letter said, if you're housebound or severely fatigued then we can't accept you because you need to travel to appointments*” (Participant 7).

Other services had referral criteria which were unclear and didn't direct participants to services which would meet their needs:

“…*and also, then I read about all the long covid clinics and they vary so much. Some actually reject people who have been referred*” (Participant 11).

“*I keep running up against brick walls trying to get into a long covid clinic, or they sort of say, oh, yeah, you've got a clear chest x-ray, you're fine. But I still can't breathe properly*” *(*Participant 5).

In some cases, referral criteria appeared to exclude those most in need of support:

“*My health authority, only offer support to people who can benefit from 6-to-8-week psychological interventions. In other words, who aren't very ill*” (Participant 2).

There was also a lack of clarity regarding the availability of services, and participants were unable to determine details such as opening dates or where else they could seek assistance. One participant was advised by her GP:

“*Apparently the long covid clinic at the hospital isn't actually open yet, its open on paper, but because of the vaccine rollout and all that, they've had to delay the opening of it. So, what we'll do is we put you on a list with a load of other people we'd also tried to refer, and as soon as it opens, we'll rerefer you*” (Participant 5).

Participants also reported incidences of people being turned away by services without advice on where they could access appropriate support:

“*One of the women in the group said she phoned—she lives alone—she phoned and said that she could barely speak. And they said Well if you can speak enough on the phone, you're not ill enough to go to hospital*” *(*Participant 6).

In some cases, when services were unavailable, or possibly because the healthcare professional was unsure of which service the patient required, participants reported being referred to services unrelated to the symptoms they were experiencing. For example, one participant requested support following a relapse:

The doctor said, “*well the only thing I can offer is physiotherapy. I thought I'll take it. I'll take anything at this point*”… but “*the physical therapist deemed it too early for any type of physical therapy*” (Participant 3).

Another participant was struggling with fatigue and breathing problems and wanted a CT scan as she had been reading research which suggested that COVID-19 could affect the vascular system. However, the cardiologist refused, saying that further investigation wasn't warranted. The cardiologist then referred the participant to a sleep clinic for fatigue. The participant was then upset, as:

“*I'm not sleepy during the day, I'm not drowsy, I just feel like I've run out of battery*” (Participant 11).

Another participant was seeking assistance with practical tasks as her fatigue meant she was unable to self-care, or manage household tasks such as laundry or food preparation. However, the services she required were not available:

“*When I first rang the council to ask for some home help, it was just someone on the phone, he'd just been hired in on a phone, and he said, is there someone to do shopping for you? I said, well, my son goes shopping. He said, oh, that's all we can offer you*” (Participant 7).

Sometimes, participants felt dismissed when they were directed to a website or other source of information on their condition, as an alternative to being seen by a health professional:

“*I had to phone back for the second results and at that point, all they did was refer me to, albeit a very good, website*” (Participant 9).

Other participants had an initial meeting with a health professional and then didn't hear anything further. Overall, the difficulty in finding supportive health service left participants feeling alone, and exhausted:

“*I, the person who's trying to manage fatigue and activity, I have to constantly chase this. They talk about treatment pathways, what are they? How do you get on them, would be a big question? How do you get on them?*” (Participant 3).

When healthcare professionals were unable to offer a recommended medical intervention, participants felt unsupported. Participants reported that they were sometimes left feeling alone in facing their health problems:

“*Eventually a doctor came and he said, yeah, your chest X-ray is clear, your bloods are clear. I was like well, if anybody had just spoken to me before you did these things, I could have saved you money and told you not to bother running these tests and running these chest x-rays, because I'd had that twice already this year, in the last 6 months, and they were all fine. Oh, well there's nothing wrong with you. Well, I was gasping for breath, and I said, yeah there must be because I can't breathe. And he said, no, there isn't anything we can help you with, there's nothing wrong with you, you're discharged, you can go home now. Well, I couldn't walk to the door to get out. I got as far as, like, I don't know, about ten yards away from the room, and somebody said, right, we'll get a chair. And they got a wheelchair and wheeled me outside and just left me there. And I couldn't breathe*” (Participant 5).

Participants were left feeling misunderstood, unable to access the help they required, and abandoned:

“*You kind of felt you were just in this batch of people that were almost collateral damage to the whole thing. It was just like, we can't deal with you because we're scared, so we're just going to leave you to die basically*” (Participant 12).

#### Theme 4: “It's Real to You”

Some participants felt that they were not believed, and that health professionals were basing their decisions upon other personal characteristics. This increased participants' feeling of frustration:

“*The words came out of his mouth of, its real to you*” (Participant 10).

This participant was also asked “*how are things at home?*” by her pulmonary doctor following her descriptions of being breathless and unable to breath normally. She felt that the severity of the difficulties she was having weren't taken seriously, or investigated properly because of her gender and age:

“*They said, oh, you've got breathing pattern disorder, don't you worry, you've just got into a bad habit with your breathing, and a bit of physiotherapy, and you'll be fine*.” … “*And I realized then that he was assuming a lot about me, without having met me before, that being a woman of a certain age is a disadvantage apparently, apparently my profession didn't help*” (Participant 10).

This left the participant feeling upset, and alone:

“*But I was really upset that they thought it was in my head. And that was definitely the worst part for me was when I came out of that appointment and just, you know, I'm on my own. There's no medical help here*” (Participant 10).

Similarly, another participant found that her doctor considered anxiety to be responsible for the symptoms she was experiencing:

“*I'm working my way through the GPs at my surgery because, I had one recently who started talking about people that take to their bed and how they fared and some people that didn't. I felt … that's made my mind up. Two started talking about positive thinking, so I thought yeah positive thinking isn't going to make me well and I'm quite positive as it is. So yeah, I think this GP who said about positive thinking, she also when I first spoke to her was saying to me, are you anxious, how's your anxiety? I said, I haven't got any. It was almost presumed to be an anxiety thing, and when its… I mean I know some people are struggling with anxiety as a result of COVID, but I have been ok*” (Participant 11).

Another participant believed that her breathing difficulties weren't investigated as the doctor thought they were caused by her being overweight:

“*I spoke to one of the doctors there, and she was actually quite rude and said, yeah, well, yeah, no, looking at your weight and height you're you know, the BMI is 30-point-something, which makes you obese, so you're probably struggling with your breathing as a result of that. And I said to her, look, I'm actually a stone and a half lighter than I was before I got COVID, I said, and I've never had problems with my breathing before, I've been heavy for a few years*.” “*I thought, oh, right, so I'm overweight, so you're going to dismiss me, that's that*” (Participant 5).

Doctors were not certain of the impact of medication on people experiencing COVID-19 and long covid, due to the newness of these conditions. This meant that some participants were unable to access treatments for particular problems:

“*I can't get my GP to prescribe me a H2 blocker which is an antihistamine, because it's not licensed for long covid. But it is licensed for reflux, which is what I've got, but it's linked to long covid. But she's like no, because it's not licensed for use in long covid. Other peoples' GPs are giving it to them. But she's sticking to the rules, basically*” (Participant 6).

In some cases, when GPs felt participants required more support, they offered referrals to services they knew were available, even if these did not always appear useful to the participant. One participant was offered social prescribing and said she responded by writing on the form:

“*What does it mean*?” (Participant 4).

Another participant received a referral to a sleep clinic when she was seeking assistance with her breathing.

The lack of knowledge regarding long covid meant that participants weren't provided with information regarding the path to recovery and were left unclear about their future health and abilities:

“*I don't know at this stage whether I can pick it up again, and I'm going to be devastated…. The thought of giving up was so really scary, because it will be down to COVID if I can't pick it up*”. “*So, I'm not somebody who wants to just sort of sit around, go out for little walks, read a book, listen to the radio, you know, whatever; it's not me*” (Participant 2).

Another participant, discussed the meaning of illness and recovery being different for each individual:

“*I remember saying to my doctor, I said, the thing is I'm not sure how ill I really am, because 3 years ago I decided to find out how far I could walk in 4 h, and the answer was 16 miles. You know, I feel it'll be a long, long, time before I'm anywhere near that level of capability*” (Participant 1).

This means that interventions and treatments have to be adapted to meet individual needs:

“*I almost need a coach to encourage me, not just once a week on a session, but on a more regular basis, to give me some goals*” (Participant 1).

One participant felt that healthcare staff failed to listen to the patient's point of view and often dismissed people because they didn't yet understand how to treat a particular condition:

“*The problem I have with the NHS, which is how would the NHS… it's the arrogance of, if we don't know the answer there isn't one or we haven't tackled it yet or you're wrong or you're imagining it and I think with ME, that's been quite a classic example*” *(*Participant 9).

#### Theme 5: Patient as Expert

Where healthcare professionals acknowledged limitations in their understanding of the illness the participant was experiencing, this facilitated a mutual working relationship between the patient and professional. In some cases, the participant was able to share the knowledge they had gained about their condition:

“*I had strong chest pain, and the young doctor who saw me had not hear of long covid. I mean, this was probably 9 months ago. I explained it to her, and she went and looked it up, because when she came back, she had a completely different take on things, and bless her for doing that, you know?*” (Participant 10).

In this study, participants demonstrated that they had developed expertise in their condition through contact with long covid support groups and reading information they found online. They discussed research they had read, conversations with experts, and were able to describe complex medical tests and clinical conditions:

“*I've found out through one of my research ladies about the role of estrogen in COVID in ladies. So, I did my homework and went to the GP and said can I have some HRT? Because COVID stopped my periods and made me menopausal*” *(*Participant 11).

Another strategy for accessing the required support involved seeking out professionals with particular interest or knowledge in long covid:

“*I asked in my GP surgery, are any of the GPs up on long covid and keeping up with it and they named this other doctor, so I was able to speak to her. But if I hadn't asked, I wouldn't have been told that*” (Participant 6).

One participant found that lockdown impacted access to services provided her with the information she needed to manage her pre-existing long-term condition:

“*Basically, all the check-ups that I have, and everything, and the monitoring, just stopped*” (Participant 8).

Participants found that strategies they previously used to manage pre-existing conditions were less effective, or exacerbated the fatigue or breathlessness associated with long covid. However, they were unable to access information about how the two conditions could be managed together:

“*I would like to know, the exercises I try to do—because I can't walk very far, I try to do exercises—are really the sort of drama and dance school exercises I learned when I did a theater studies degree when I left school, and then taught; and I use that, and it's very much Pilates based and stretching and all that. But I don't know whether I'm doing the things that are most useful, because the pain that I've got with the long covid, which is different, it's not making the neuritis worse it's just shooting pains everywhere, and that is quite severe. And you know, some kind of help with things like that, some kind of help basically with just coping … and what I can't do*” (Participant 2).

In some cases, healthcare services successfully supported participants to manage their own condition. One participant described receiving rehabilitation for cardiac problems following COVID, and found that after a 12-week course, he was able to manage his own recovery program:

“*So, the whole service disengaged with me… not that long ago, a couple of months ago. It doesn't bother me that they're not supporting me* ‘*cause all the notes they'd given me and the links to the British Heart Foundation*,' *I've got. So, if I'm ever not sure, I can check it out*” (Participant 1).

Participant expertise was also recognized in other areas, and one participant was asked by her manager to provide information which would assist them to support other staff members experiencing long covid, in their return to work.

#### Theme 6: Making Connections

Participants with long covid reported that they found that engagement with other people who were experiencing the same condition provided them with a sense of universality which was therapeutic:

“*Having that connection to other people who are also going through it it's really, really been the most important thing, because as frustrated and upset as I am, they are as well, and that makes me feel better*” (Participant 11).

“*The most help I've had has been from these peer support groups*” (Participant 5).

One participant spoke of the relief she felt when an online survey asked questions which related to how she was feeling, for the first time:

“*And just to be asked that, do you have an unusual tightness in your chest? Really, that was so comforting, which sounds really odd. And it wasn't even a real person asking me, it was just a survey*” (Participant 10).

The pandemic situation meant that everyone experienced restrictions on their freedom to visit friends, family and other sources of support. One participant had shielded throughout the pandemic, following medical advice, due to pre-existing health conditions. The participant found being unable to spend time with friends and family was very difficult, particularly as her granddaughter was also experiencing a healthcare crisis at this time:

“*I wasn't feeling well anyway, with physical health problems and I was getting more and more depressed*” (Participant 8).

The participant relied on her partner for shopping, collecting prescriptions and other essential tasks outside the home. However, she found that having to remain at home had a profound effect on her mental health:

“*I had a psychologist for telephone appointments to talk about.because my depression at one point was so bad, I thought I just had to go to Dignitas and just get out of this*” (Participant 8).

#### Theme 7: Thoughts About the Future

Overall, participants were frustrated with the difficulties they had experienced in accessing healthcare during the pandemic. They were fearful that their condition was never going to improve, and that they would be unable to return to their former vocational and leisure activities:

“*I'd like to have the proper care, to know whether I have got some damage in my body that either can be managed, but so then I know what I'm dealing with, or can be treated, or managed, and then I know*” (Participant 13).

Participants were also concerned that the continuing COVID-19 pandemic would leave many more people in a situation similar to the one they were experiencing, and pleaded for action to be taken urgently:

“*Look, this is going to be a nightmare going forward, and you're either going to ignore it and its going to be a nightmare for a hell of a lot of people who aren't going to be able to work and who won't be able to get help, and will basically be disabled, so economically and health-wise, NHS-wise, it's going to be a nightmare. Or you actually deal with it now and get these things set up now and tell people that we're open again*” (Participant 5).

## Discussion

The ultimate goal of person-centered healthcare is to create a healthful culture based upon respect for the individual. Findings indicate that participants have not always experienced this during the COVID-19 pandemic. This study examined the experiences of people who were unwell and experienced feelings of isolation during the COVID-19 pandemic, using a framework based on the Person-centered Practice Framework (PcPF) (21).

The participants interviewed for this study were mainly female, and were recruited from an online survey, where again, the respondents were mainly female. The reasons for this are unclear, but may include women, in addition to older people, are more likely to experience long covid (27, 28). Previous research has also found that women may have a different experience of healthcare services from men. As indicated by findings from this study, women are more likely than men to experience delays in diagnosis, and to have difficulty accessing health services (29). Further, previous research found women were more likely to be misdiagnosed with depression, possibly by as much as 30–50%, which ironically, may increase the levels of stress or depression experienced by women (29).

In some cases, participants felt clinicians believed they were hysterical or exaggerating their condition, which prevented the investigation of reported symptoms. Other participants felt their clinician was unwilling to look beyond them being overweight or possibly approaching menopause, even though they felt this did not account for all the symptoms they were experiencing. These findings indicate that participants may have experienced the effects of unintentional cognitive biases based upon cultural stereotypes (30).

Components of the PcPF support effective communication; exploring the illness experience; understanding the patient as a whole person; and finding common ground (31). Generally, participants who were already familiar with their GP, reported a higher level of person-centered interaction. However, participants felt their symptoms were often dismissed by health professionals, possibly because limited information regarding long covid, prevented professionals from arriving at this diagnosis. Alternative explanations may include increased inequity due to discrimination; and poverty lead to women experiencing a greater degree of ill health than was seen in their male counterparts (32).

Female participants may have experienced particular adverse effects relating to illness during the pandemic due to the non-negotiable responsibilities traditionally held by women. For example, women often assume more responsibility for household tasks, and childcare, or other unpaid caregiving roles. In Scotland, 59% of people who provide unpaid care to a relative, friend or neighbor are women (33). Findings suggest that lockdown policies, which prevented people from different households meeting, also increased difficulty in accessing support from family and friends, leaving people to manage these tasks alone. Health professionals appear not always to have been able to consider individual needs and priorities, and participants were unable to access appropriate interventions or practical support (34).

Additionally, findings indicate that participants were unable to follow long covid management advice to rest and pace their activity, due to work commitments and childcare responsibilities. Women are more likely to be in work that is poorly paid, on short-term or 0-h contracts, which are less likely to offer support for people who are unwell and unable to work (35). Participants struggled with managing childcare, household tasks and paid work and were not able to access support in these areas. Rest and pacing are more difficult for people who are not receiving support in terms of paid sick leave, flexible working hours, or reduced workload.

Limited understanding of long covid, combined with variable access to healthcare services as a result of pandemic restrictions may also have resulted in people seeking support for symptoms at a later stage. Study participants felt that pre-existing conditions may also have been less effectively managed due to limited access to support services and medical checks. Alternatively, they may have missed the reassurance of face-to-face meetings and examinations (36). In some cases, such delays can be associated with adverse outcomes (37).

Good healthcare requires up-to-date professional knowledge and skills (38). Clinical skills to manage people with long covid, include “listening to the patient, documenting what the symptoms are, how they change and how they fluctuate, and being alert to symptoms that might suggest they need referring” (8). Participant reflections from this study suggest that this was not always the case, however, and some GPs did not fully consider their point of view, supporting the results from a previous study (36).

These findings suggest that in some cases, participants felt dismissed by health professionals, or that they weren't properly listened to, resulting in the professional making assumptions regarding their situation. In addition to demonstrating the previously described clinical skills, it is important for healthcare professionals to develop the prerequisites for person-centered healthcare of “knowing self.” Health professionals need to be self-reflexive and willing to challenge their own preconceptions or unconscious biases regarding relationships between personal characteristics such as gender, and health. Reflexive learning should be provided to ensure that all health professionals are able explore their responses to their patients, impacts of their reactions, and consider alternative responses (39).

The absence of a formal definition for long covid seems to have impacted negatively on the recognition of their participants' symptoms by some healthcare professionals and obstructed treatment, although many of the associated conditions e.g., chronic fatigue syndrome, were familiar. This in turn, may have triggered further psychological impact for people (40). Findings from this study support previous research which identified negative mental health impacts from the pandemic (41).

Where professionals took time to listen, and demonstrated empathy, participants were more likely to feel supported, and in some cases formed alliances with the healthcare professional, where participants and health professionals could work together to investigate possible treatment interventions and opportunities. Participants reported sharing information including research papers and articles related to their condition with healthcare professionals. This indicated that felt they were working in partnership with the health professional, and that their own concerns, or interest in their condition was shared. These experiences supported the value to participants of person-centered processes—particularly engaging with the persons' beliefs and values, being sympathetically present, and working in partnership. This engagement contributed to participants feeling supported even when the healthcare professional was unable to provide expert knowledge, or access to interventions or services which could assist in the participant recovery.

Conversely, when participants felt dismissed or misunderstood, they reported increased feelings of frustration or isolation. Lockdown, and a focus on acute care services to mitigate pandemic related mortality, meant that people who were shielding lost access to services which were required to monitor and maintain their health. Hence, at a time of particular stress related to the pandemic, people were increasingly concerned that their condition was not being managed effectively. As many clinics were closed, participants were often unable to engage in shared decision-making regarding the relative risks of attending clinics during a pandemic, or reduced monitoring of their condition. Some healthcare services also appear to have been slow to offer opportunities for patients to attend consultations online or by telephone. Additionally, some participants sought face-to-face meetings, particularly for periodic reviews of their condition which required medical tests, or to discuss complex issues.

Findings from this study support the view that effective clinical skills include building a professional caring relationship which recognizes the concerns of the patient. Healthcare professionals can be supported in building effective person-centered relationships by organizational systems which facilitate person-centered relationships by offering people the opportunity to book appointments with a selected GP, and appointment times should allow sufficient flexibility for the healthcare professional to be able to consider the personal beliefs and values of the individual. Additionally, systems should be reviewed reducing the impact of unconscious bias. Findings also suggest increasing direct patient access to specialist services may provide a more streamlined service- reducing GP burden, and patient waiting times (37).

Participants found that support groups provided useful information on access to tests and interventions, but also offered authentic relationships and a level of understanding that most participants were unable to access elsewhere. Social support interventions can unite people with a common experience, creating a sense of belonging and meaningful connection, as well as increasing individual competence and perceived ability to cope (42). Participants who were able to access this type of group found sources of social support, and information on their condition. They were also able to share positive experiences from their own journey providing support and hope for others. Despite these benefits, only one participant mentioned that they had found this type of group through information provided by their healthcare professional.

The NHS is organized according to medical specialties, and services often aim to manage single-conditions. During the pandemic, existing services redirected their focus from chronic conditions, toward interventions for acute COVID-19. Participants were unable to access information regarding long covid, or interactions between long covid and any pre-existing condition they experienced. Narrow referral criteria and geographical service variations, often limited access to professional services with expertise which was sought by the participant. Alternatively, participants were referred to multiple services for assessment of each of the physical systems affected by long covid resulting in fragmented care from a number of different specialties (43). To support people to manage their own health conditions, there needs to be flexibility which facilitates access to services they have identified as essential. This outcome recognizes the individual as the expert, and healthcare services work in partnership to support them. By placing the person at the center of services, barriers between different providers can be broken down.

### Limitations

Due to the rapid nature of this study, limited numbers of participants were interviewed (*n* = 11). This small sample size means that findings are not transferable or generalizable, and further research is required to validate results for the wider population (44). Participants were mostly female (*n* = 10), and all but one participant, experienced symptoms of long covid (*n* = 10). All participants were resident in the UK. Participants were recruited following completion of an online survey concerning access to support for people who were unwell during the pandemic, which was promoted on social media. This study focused on the point of view of people of a small population who had been unwell during the covid-19 pandemic and had difficulty accessing support. It did not explore the impact of the pandemic from the point of view of people providing healthcare services.

## Conclusions

In summary, this study examined the views of people who were unwell during the COVID-19 pandemic. Participants reported feeling dismissed, and abandoned by healthcare services, and struggled to access services which offered understanding of their condition, or support with the tasks they were unable to manage. The Person-centered Practice Framework offers a useful lens for understanding difficulties faced by people attempting to access care. We have found that people want to feel heard, supported to navigate healthcare systems, source trustworthy information, find appropriate services, and collaborate in learning and problem-solving with healthcare professionals. Moving forward is crucial, with greater emphasis on overcoming barriers to person-centered healthcare. This should focus on steps now and also in planning for the possibility of further rapid changes in the demand for and provision of healthcare.

## Data Availability Statement

The datasets presented in this article are not readily available because the dataset contains participant identifiable data. Requests to access the datasets should be directed to cbulley@qmu.ac.uk.

## Ethics Statement

The studies involving human participants were reviewed and approved by Queen Margaret University Ethics Review Committee, Queen Margaret University, Edinburgh EH21 6UU. Written informed consent for participation was not required for this study in accordance with the national legislation and the institutional requirements.

## Author Contributions

CB, VT, LS, RM, OS, KN, KS, BM, and JD contributed to conception and design of the study. CB led the study. VT developed the online survey and organized the database. EC performed the interviews and thematic analysis. EC and CB wrote the first draft of the manuscript. All authors contributed to manuscript revision, read, and approved the submitted version.

## Conflict of Interest

The authors declare that the research was conducted in the absence of any commercial or financial relationships that could be construed as a potential conflict of interest.

## Publisher's Note

All claims expressed in this article are solely those of the authors and do not necessarily represent those of their affiliated organizations, or those of the publisher, the editors and the reviewers. Any product that may be evaluated in this article, or claim that may be made by its manufacturer, is not guaranteed or endorsed by the publisher.
